# A Comparative Study of Moral, Emotional, and Spiritual Intelligence in Parents of Aggressive and Non‐Aggressive Children: A Developmental Psychology and Parenting Behavior Perspective

**DOI:** 10.1002/brb3.71404

**Published:** 2026-04-14

**Authors:** Mahdi Naeim, Mohammad Narimani, Niloofar Mikaeili, Saghar Seifi

**Affiliations:** ^1^ Department of Psychology, Faculty of Educational Sciences and Psychology University of Mohaghegh Ardabili Ardabil Iran; ^2^ Department of Psychology Islamic Azad University, Ardabil Branch Ardabil Iran

**Keywords:** aggression, emotional development, emotional intelligence, moral intelligence, parenting, spiritual intelligence

## Abstract

**Objective:**

This study aimed to compare levels of moral, emotional, and spiritual intelligence in parents of aggressive versus non‐aggressive children.

**Methods:**

A causal‐comparative design was employed. The study population included parents of elementary school students in Ardabil, Iran. Using a multistage sampling method, 60 participants were selected. Research instruments included the Shahim Aggression Questionnaire (2006), Lennick and Kiel's Moral Intelligence Questionnaire (2011), Bar‐On Emotional Intelligence Questionnaire (1997), and King's Spiritual Intelligence Questionnaire (2008). Data were analyzed using multivariate analysis of variance (MANOVA). Basic demographic information was collected, and sampling procedures aimed to reduce socioeconomic variability between groups.

**Results:**

MANOVA revealed a significant overall group difference across parental moral, emotional, and spiritual intelligence; *F*(3, 58) = 27.10, *p* < 0.001, *η* ^2^ = .421. Parents of aggressive children showed lower mean scores across all domains, with the largest difference in emotional intelligence (*M* = 2.21 vs. 3.11). Follow‐up univariate analyses confirmed significant group differences for moral intelligence, *F*(1, 58) = 61.43, *p* < 0.001; emotional intelligence, *F*(1, 58) = 78.64, *p* < 0.001; and spiritual intelligence, *F*(1, 58) = 56.34, *p* < 0.001. These results indicate associations between parental intelligence dimensions and child aggression, without implying causal direction.

**Conclusions:**

The findings highlight that parental moral, emotional, and spiritual intelligence are linked to differences in children's aggressive behavior. These results suggest that parent‐focused interventions, such as programs to enhance emotional regulation, ethical decision‐making, and meaning‐oriented coping skills, may help parents strengthen their interactions with children, supporting adaptive behaviors and potentially reducing maladaptive outcomes.

## Graphical abstract

This study reveals that parents of aggressive children exhibit significantly lower moral, emotional, and spiritual intelligence compared to parents of non‐aggressive children, with the widest gap in emotional intelligence. These findings suggest parent‐focused interventions enhancing these domains may help reduce child aggression.

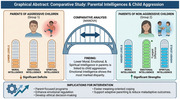

## 1 Introduction

Childhood aggression is one of the major challenges in developmental psychology and mental health, with widespread negative consequences at both individual and societal levels (Verhoef et al. 2022; Pu and Rodriguez 2021). Children who exhibit aggressive behaviors are more likely to experience peer rejection, academic difficulties, heightened risk of anxiety and depression, and, eventually, the development of personality disorders in adulthood (McRae et al. 2021). These children often struggle with social skills, conflict resolution, and self‐regulation, and in the absence of timely interventions, their behavioral problems may persist and become chronic (Meijer et al. 2024; Beckmann et al. 2021). Preventing and identifying the factors associated with childhood aggression in early life is of particular importance, given the high neuropsychological plasticity during this developmental period (Ip et al. 2021; Shahim 2007).

Among the most critical factors influencing the emergence of aggression in children is the family environment, particularly parental characteristics (Navarro et al. 2022; Li et al. 2024). Parents play a fundamental role in shaping emotional regulation, behavior, and social skills in their children. While previous research has extensively focused on parenting styles, such as harsh discipline, permissiveness, or corporal punishment, few studies have explored the internal psychological traits of parents, including various forms of parental intelligence (Goagoses et al. 2023; Ndengeyingoma et al. 2024).

In this context, three specific dimensions of parental intelligence, emotional intelligence, moral intelligence, and spiritual intelligence, may play a pivotal role in regulating children's behaviors and preventing aggression.

Emotional intelligence involves the ability to identify and understand emotions, empathize with others, regulate negative emotional states, and express feelings in adaptive ways (Filice and Weese 2024). Parents with higher emotional intelligence are typically better equipped to manage aggressive behaviors in their children by modeling emotional control and providing constructive emotional guidance (Lau and Williams 2022).

Moral intelligence refers to the capacity to distinguish right from wrong and to act by ethical values, encompassing dimensions such as integrity, responsibility, forgiveness, and compassion (Beiranvand and Yaghoobi 2024). Parents with higher moral intelligence tend to interact with their children in a fair, consistent, and empathetic manner, which can reduce the likelihood of problematic behaviors, including aggression (Essler and Paulus 2022).

Spiritual intelligence involves the ability to find meaning and purpose in life, accept difficult experiences, and connect with transcendent aspects of existence (Amram 2022; Pinto et al. 2024). Parents with higher levels of spiritual intelligence are often more patient, balanced, and accepting in their parenting practices. This creates a nurturing and calm environment that reduces the probability of aggressive behaviors in children.

### 1.1 Conceptual and Theoretical Framework

Moral intelligence is conceptually defined as the capacity to distinguish right from wrong and to behave in accordance with ethical principles such as integrity, responsibility, compassion, and forgiveness. Lennick and Kiel (2011) conceptualized moral intelligence as a practical form of ethical competence that guides decision‐making and social behavior. From a developmental perspective, moral functioning in parents influences children through modeling, reinforcement, and value transmission, shaping behavioral regulation and prosocial tendencies (Essler and Paulus 2022).

Emotional intelligence refers to the ability to perceive, understand, regulate, and express emotions effectively in oneself and others. According to the ability‐based model proposed by Mayer et al. (2004), emotional intelligence plays a central role in adaptive social functioning and emotional self‐regulation. In parenting contexts, emotionally intelligent caregivers provide emotional scaffolding that helps children develop regulation skills and reduces impulsive or aggressive responses (Lau and Williams 2022).

Spiritual intelligence encompasses higher‐order cognitive and existential capacities that enable individuals to construct meaning, maintain existential awareness, and integrate transcendent perspectives into everyday life. King (2008) described spiritual intelligence as a set of mental abilities supporting reflection, purpose, and adaptive coping. Empirical work suggests that spiritually oriented meaning‐making promotes emotional balance, patience, and reflective parenting practices that buffer stress‐related reactivity (Pinto et al. 2024).

Although these forms of intelligence are conceptually distinct, theoretical perspectives suggest meaningful interconnections among them. Emotional regulation supports ethical judgment and moral action, while spiritual meaning‐making enhances resilience and value‐consistent behavior. Together, these capacities form a multidimensional framework of parental psychological competence that influences how parents interpret, regulate, and respond to child behavior. Examining moral, emotional, and spiritual intelligence simultaneously therefore provides a comprehensive theoretical lens for understanding parental factors associated with childhood aggression.

Despite the recognized importance of these forms of parental intelligence in child development, most existing studies have examined these variables separately. To date, limited research has simultaneously investigated emotional, moral, and spiritual intelligence in parents of aggressive and non‐aggressive children. This gap in the literature limits our understanding of how these types of intelligence may interact and collectively influence child behavior.

The present study aims to address this gap by comparing the levels of emotional, moral, and spiritual intelligence in parents of aggressive versus non‐aggressive children. This comprehensive approach may provide a valuable foundation for designing educational programs and psychological interventions to reduce childhood aggression.

## 2 Method

### 2.1 Study Design

This study employed a causal‐comparative (ex post facto) design. The primary focus was to examine differences in parental levels of moral, emotional, and spiritual intelligence based on whether their children exhibited aggressive or non‐aggressive behaviors. In this design, child aggression served as the dependent variable, while parents' moral, emotional, and spiritual intelligence were considered independent variables.

### 2.2 Participants and Sampling

The target population of this study consisted of all male elementary school students in Ardabil, Iran. According to official enrollment data, during the 2024 academic year, 51,213 students were attending 245 elementary schools in the city.

A multistage random sampling procedure was employed to select participants. In the first stage, 10 schools were randomly selected from the list of elementary schools. In the second stage, a proportional number of students were randomly chosen from each selected school. The sample size was determined based on table, which provides guidelines for selecting an appropriate sample from a given population, resulting in an initial sample of 382 students.

To assess levels of aggression, teachers completed the Shahim Aggression Questionnaire for each student. Following this assessment, two groups were identified based on the questionnaire scores: one group included students with high aggression scores (aggressive group), and the other included students with low aggression scores (non‐aggressive group).

For the final analysis, 60 parents were selected, consisting of the parents of 30 aggressive children and 30 non‐aggressive children.

### 2.3 Demographic Variables and Control of Confounders

Basic demographic information including parental age, education level, and family structure was collected to provide contextual insight into the sample characteristics. Efforts were made during sampling to select participants from similar school districts to reduce variability associated with socioeconomic background. Although the study design did not involve formal statistical control of all potential confounding variables, the grouping procedure aimed to ensure relative comparability between parents of aggressive and non‐aggressive children. These demographic considerations were taken into account when interpreting the findings.

The final sample size of 60 participants (30 per group) is consistent with sample ranges commonly used in causal‐comparative psychological research. Furthermore, the large observed effect sizes suggest that the sample provided sufficient statistical sensitivity to detect meaningful group differences.

### 2.4 Instruments

#### 2.4.1 Shahim Aggression Questionnaire (2007)

This 21‐item questionnaire measures two forms of aggression: overt and relational, and assesses three main components: physical aggression, verbal aggression, and relational aggression. Its reliability and validity have been confirmed in multiple studies. In the current sample, internal consistency was assessed, yielding a Cronbach's alpha of 0.88, indicating good reliability (Shahim 2007).

#### 2.4.2 Lennick and Kiel's Moral Intelligence Questionnaire (2011)

This 40‐item questionnaire evaluates four dimensions of moral intelligence: integrity, responsibility, forgiveness, and compassion. Its reliability has been reported in Iranian samples with a Cronbach's alpha of 0.89. In this study, Cronbach's alpha for the total scale was 0.91, confirming acceptable internal consistency in the parent population (Raisi et al. 2018).

#### 2.4.3 Bar‐On Emotional Intelligence Questionnaire (1997, Short Form)

The short form consists of 30 items assessing self‐awareness, empathy, emotional regulation, and social skills. In the current sample, Cronbach's alpha was 0.87, supporting reliability among Iranian parents (Vahdat‐lasemi et al. 2024; Bar‐On 1997).

#### 2.4.4 King's Spiritual Intelligence Questionnaire (2008)

This 29‐item questionnaire measures four dimensions: critical existential thinking, personal meaning production, transcendental awareness, and conscious state expansion. In this sample, Cronbach's alpha was 0.90, indicating high internal consistency (Sharif Nia et al. 2015).

### 2.5 Procedure

After selecting the schools and student sample, teachers completed the aggression questionnaires for each student. Based on the scores, parents of children identified as either aggressive or non‐aggressive were invited to participate in the study. Following informed consent, parents completed self‐report measures of moral intelligence, emotional intelligence, and spiritual intelligence.

### 2.6 Data Analysis

Data were analyzed using multivariate analysis of variance (MANOVA) to examine differences between the two parental groups in terms of moral, emotional, and spiritual intelligence. All statistical analyses were performed using SPSS software.

### 2.7 Ethical Considerations

All ethical standards were strictly observed throughout the study. Participation was entirely voluntary, and informed consent was obtained from all parents and teachers after they were fully briefed on the study objectives. Data confidentiality and anonymity were strictly maintained, and all information was used solely for scientific and research purposes. The study protocol was reviewed and approved by the Research Ethics Committee of Islamic Azad University, Ardabil Branch

## 3 Results

This section presents the findings related to the comparison of moral intelligence, emotional intelligence, and spiritual intelligence between parents of aggressive and non‐aggressive children. Data were analyzed using descriptive statistics, Levene's test for homogeneity of variances, MANOVA, and univariate analysis of variance (ANOVA).

### 3.1 Descriptive Statistics

Table 1 summarizes the means and standard deviations of moral intelligence, emotional intelligence, and spiritual intelligence in the two parental groups.

**TABLE 1:** Means and standard deviations of parental intelligence variables in aggressive and non‐aggressive groups.

Variable	Aggressive group (M ± SD)	Non‐aggressive group (M ± SD)
Moral intelligence	2.25 ± 0.352	3.16 ± 0.383
Emotional intelligence	2.21 ± 0.361	3.11 ± 0.342
Spiritual intelligence	2.34 ± 0.411	3.20 ± 0.341

**FIGURE 1:**
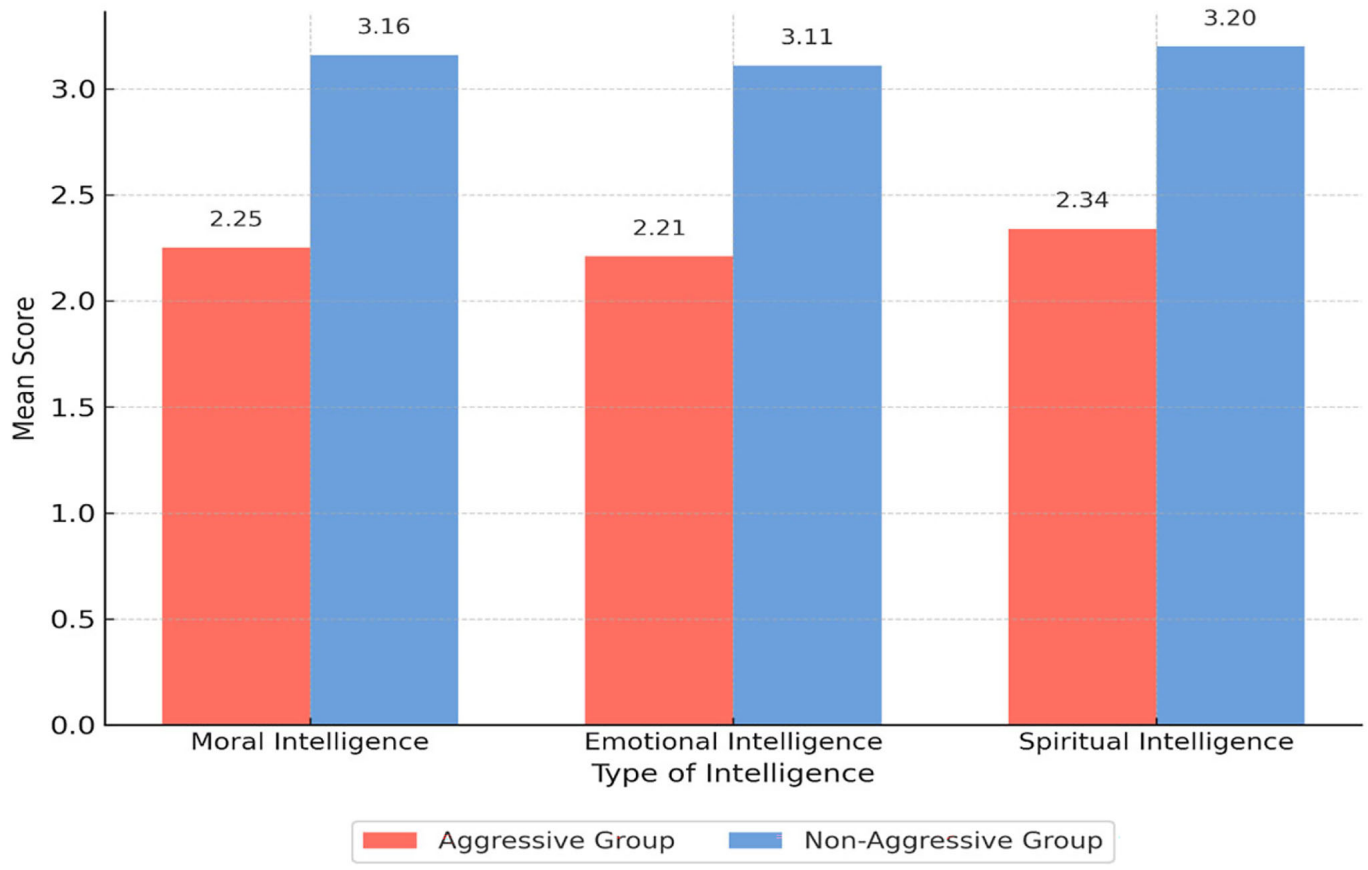
Comparison of mean scores of parental moral, emotional, and spiritual intelligence in aggressive and non‐aggressive child groups.

As shown in Table 1, parents of non‐aggressive children scored higher in all three types of intelligence compared to parents of aggressive children. The largest difference between the two groups was observed in emotional intelligence, with a mean score of 2.21 in the aggressive group and 3.11 in the non‐aggressive group. A visual comparison of these means is provided in Figure 1.

Figure 1 illustrates the mean scores of moral, emotional, and spiritual intelligence in parents of aggressive and non‐aggressive children. The non‐aggressive group showed consistently higher levels across all three types of intelligence, with the largest difference observed in emotional intelligence.

### 3.2 Levene's Test for Homogeneity of Variances

To ensure the assumption of variance homogeneity was met, Levene's test was conducted. The results are presented in Table 2.

**TABLE 2:** Levene's test for equality of variances.

Variable	*F*	df1	df2	*p*‐value
Moral intelligence	1.44	1	58	0.234
Emotional intelligence	1.48	1	58	0.143
Spiritual intelligence	1.62	1	58	0.208

Levene's test results indicate no significant differences in variance between the two groups for any of the intelligence variables (p > 0.05). Therefore, the assumption of homogeneity of variances was met, justifying the use of parametric tests such as MANOVA and ANOVA.

### 3.3 Multivariate Analysis of Variance

A MANOVA was performed to examine the overall differences between the two groups across the three intelligence variables. The results are shown in Table 3.

**TABLE 3:** Multivariate analysis of variance (MANOVA) results.

Test statistic	Value	*F*	Hypothesis *df*	Error *df*	*p*‐value	Eta squared
Pillai's Trace	0.834	27.10	3	58	0.000	0.421
Wilks’ Lambda	0.166	27.10	3	58	0.000	0.421
Hotelling's Trace	5.019	27.10	3	58	0.000	0.421
Roy's Largest Root	5.019	27.10	3	58	0.000	0.421

The MANOVA results indicate a significant overall difference between the two parental groups across the combined intelligence variables (*p* < 0.001). The Eta squared value of 0.421 suggests that 42.1% of the variance in the dependent variables is explained by group membership (i.e., whether the child is in the aggressive or non‐aggressive group).

### 3.4 Univariate ANOVA

To assess each variable separately, univariate ANOVA tests were conducted. The results are presented in Table 4.

**TABLE 4:** Univariate ANOVA results.

Variable	SS	*df*	MS	*F*	*p*‐value	Eta squared
Moral intelligence	897.800	1	897.800	61.428	0.000	0.441
Emotional intelligence	1428.050	1	1428.050	78.644	0.000	0.787
Spiritual intelligence	432.000	1	432.000	56.344	0.000	0.417

**FIGURE 2:**
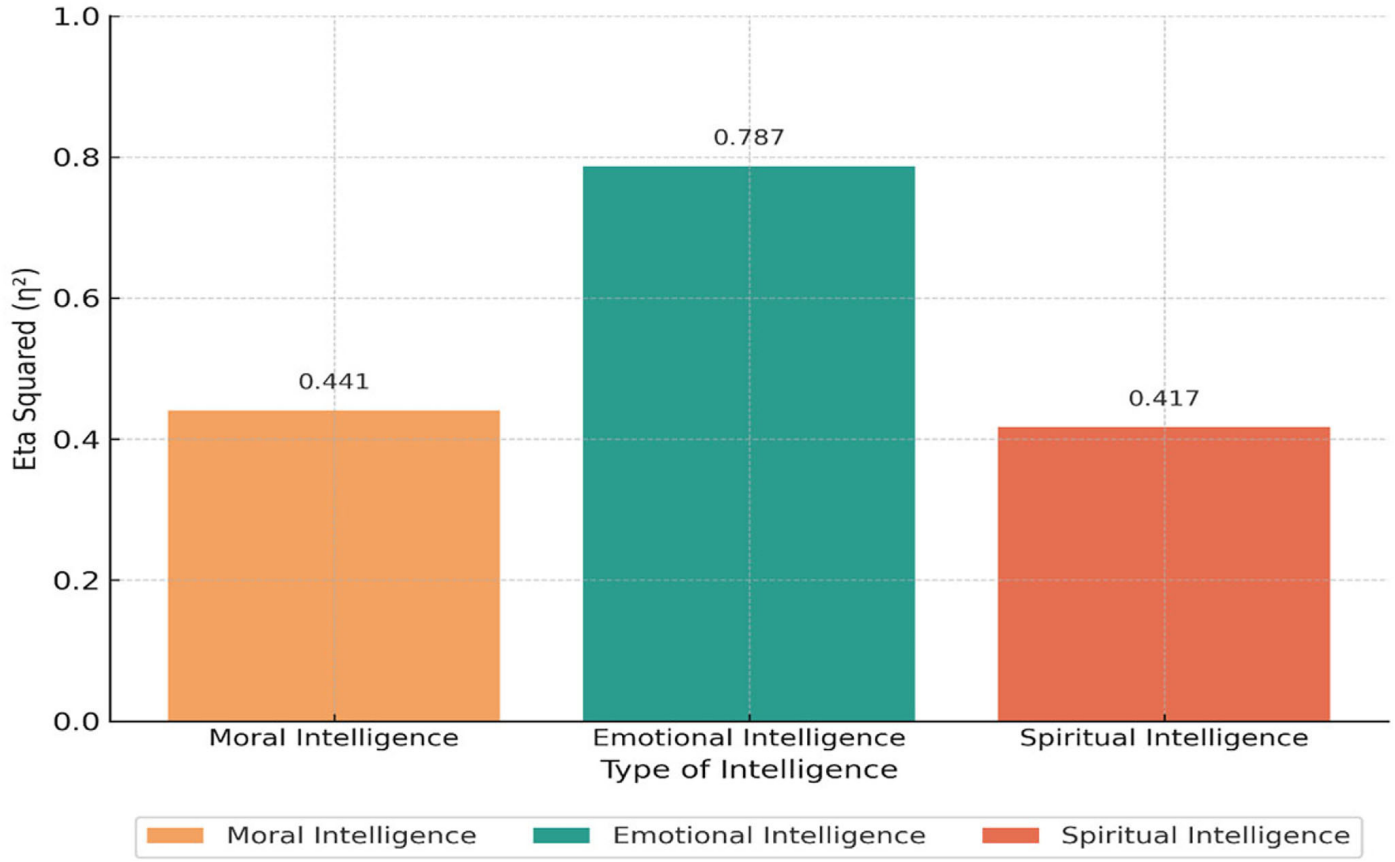
Effect sizes (Eta Squared) for group differences in parental intelligence variables.

As shown in Table 4, significant differences were found between the two groups in all three intelligence variables (*p* < 0.001). The highest effect size was observed for emotional intelligence (Eta squared = 0.787), indicating that group membership accounted for a substantial proportion of the variance in this variable. Figure 2 provides a visual summary of the effect sizes for each variable.

Figure 2 illustrates the Eta squared values for each of the three intelligence variables, indicating the proportion of variance explained by group differences. Emotional intelligence showed the largest effect size (*η* ^2^ = 0.787), followed by moral intelligence (*η* ^2^ = 0.441) and spiritual intelligence (*η* ^2^ = 0.417).

## 4 Discussion

The primary aim of this study was to compare levels of moral intelligence, emotional intelligence, and spiritual intelligence between parents of aggressive and non‐aggressive children. The findings revealed that parents of aggressive children scored significantly lower across all three domains compared to parents of non‐aggressive children. MANOVA confirmed an overall significant difference between the two groups, and univariate analyses (ANOVAs) further demonstrated that each of the three intelligence variables contributed independently to this difference. Among these, emotional intelligence accounted for the largest proportion of variance, with an Eta squared value of 0.787, indicating a substantial effect size.

These results indicate that parental intelligence, particularly emotional, moral, and spiritual dimensions, is associated with differences in children's behaviors. Parents with lower emotional intelligence in this sample were associated with higher observed levels of aggressive behaviors in their children, although the cross‐sectional, causal‐comparative design does not allow conclusions about causality (Lau and Williams 2022). The findings of the current study are consistent with previous research highlighting the significant relationship between parental emotional intelligence and children's adaptive and maladaptive behaviors (Lau and Williams 2022; Rademacher et al. 2025).

In addition, the study found that parents of aggressive children scored significantly lower in moral intelligence. These differences were associated with variations in children's behavior. This finding aligns with studies by Essler and Paulus (2022) and Dacka (2025), who emphasized that parents with higher moral intelligence model prosocial behaviors, anger management, and social responsibility. However, causal inferences cannot be drawn from the present design.

The results also indicated that parents of aggressive children scored lower in spiritual intelligence, particularly in the subcomponents of conscious state expansion and critical existential thinking. These components reflect the capacity to perceive challenging life situations from a broader, more transcendent perspective. In this study, lower spiritual intelligence in parents was associated with differences in children's aggressive behaviors. This pattern is consistent with the findings of Iqbal and Loona (2023) and Angmo and Singh (2024), who demonstrated that spiritual intelligence fosters emotional balance in parents and supports more patient, compassionate parenting.

Despite the meaningful contributions of this study, several limitations should be acknowledged when interpreting the findings. First, reliance on self‐report questionnaires may introduce response biases, including social desirability effects and subjective self‐evaluation. Second, the study did not include formal statistical control of all potentially confounding demographic variables, such as socioeconomic status, parental education, and family structure. Although sampling procedures aimed to reduce variability between groups, unmeasured background factors may still have influenced the results. Additionally, the relatively modest sample size may limit statistical power and restrict the generalizability of the findings. Finally, the sample was confined to parents of male elementary school students in Ardabil, Iran, which raises questions about the applicability of the results to female children or to families from different cultural and socioeconomic contexts. It is important to note that the causal‐comparative design of this study does not allow conclusions about causality; observed differences reflect associations only.

Notwithstanding these limitations, the findings provide important practical implications. The observed associations between parental emotional, moral, and spiritual intelligence and child aggression suggest that parent‐focused educational interventions may serve as a valuable preventive strategy. Integrating training programs that enhance emotional regulation, ethical decision‐making, and meaning‐oriented coping skills into parenting workshops could strengthen parent–child relationships and be associated with reductions in maladaptive behavioral outcomes in children.

Future research should employ longitudinal and multi‐method designs to better clarify potential causal pathways between parental psychological characteristics and child aggression. Studies incorporating larger and more demographically diverse samples are needed to improve generalizability and statistical robustness. Moreover, experimental investigations evaluating the effectiveness of targeted parental intelligence training programs would help determine whether strengthening these competencies is associated with reductions in aggressive behavior among children.

## 5 Conclusion

The findings of this study revealed that parents of aggressive children exhibit significantly lower levels of emotional intelligence, moral intelligence, and spiritual intelligence compared to parents of non‐aggressive children. These results highlight the critical role of parental psychological characteristics, particularly the ability to perceive and regulate emotions, adherence to moral values, and meaning‐making in life, in shaping children's behavioral outcomes. Given the substantial contribution of emotional intelligence to group differences, educational interventions aimed at enhancing parental emotional, moral, and spiritual intelligence could serve as effective strategies for preventing and reducing aggressive behaviors in children. Therefore, the development of parent training programs that include emotional, moral, and spiritual intelligence skills should be prioritized in counseling and psychological services for children and families.

## Author Contributions

Mahdi Naeim, Mohammad Narimani, Niloofar Mikaeili, and Saghar Seifi contributed to the conception and design of the study. Mahdi Naeim and Mohammad Narimani performed data collection and statistical analyses. Niloofar Mikaeili and Saghar Seifi contributed to the interpretation of data and manuscript drafting. All authors critically revised the manuscript for important intellectual content and approved the final version for submission.

## Funding

The authors have nothing to report.

## Conflicts of Interest

The authors declare no conflicts of interest.

## Data Availability

The data that support the findings of this study are available from the corresponding author upon reasonable request. Due to ethical considerations and privacy restrictions, the data are not publicly available.
